# Scaling-Up Exclusive Breastfeeding Support Programmes: The Example of KwaZulu-Natal

**DOI:** 10.1371/journal.pone.0002454

**Published:** 2008-06-18

**Authors:** Chris Desmond, Ruth M. Bland, Gerard Boyce, Hoosen M. Coovadia, Anna Coutsoudis, Nigel Rollins, Marie-Louise Newell

**Affiliations:** 1 Human Sciences Research Council, Durban, South Africa; 2 Africa Centre for Health and Population Studies, University of KwaZulu-Natal, Somkhele, South Africa; 3 Division of Developmental Medicine, Glasgow University, Glasgow, United Kingdom; 4 Centre for HIV/AIDS Networking, University of KwaZulu-Natal, Somkhele, South Africa; 5 Department of Paediatrics and Child Health, University of KwaZulu-Natal, Somkhele, South Africa; 6 Centre for Paediatric Epidemiology and Biostatistics, Institute of Child Health, University College, London, United Kingdom; Institute of Clinical Effectiveness and Health Policy, Argentina

## Abstract

**Background:**

Exclusive breastfeeding (EBF) for six months is the mainstay of global child health and the preferred feeding option for HIV-infected mothers for whom replacement feeding is inappropriate. Promotion of community-level EBF requires effective personnel and management to ensure quality counselling and support for women. We present a costing and cost effectiveness analysis of a successful intervention to promote EBF in high HIV prevalence area in South Africa, and implications for scale-up in the province of KwaZulu-Natal.

**Methods and Findings:**

The costing of the intervention as implemented was calculated, in addition to the modelling of the costs and outcomes associated with running the intervention at provincial level under three different scenarios: full intervention (per protocol), simplified version (half the number of visits compared to the full intervention; more clinic compared to home visits) and basic version (one third the number of visits compared to the full intervention; all clinic and no home visits). Implementation of the full scenario costs R95 million ($14 million) per annum; the simplified version R47 million ($7 million) and the basic version R4 million ($2 million). Although the cost of the basic scenario is less than one tenth of the cost of the simplified scenario, modelled effectiveness of the full and simplified versions suggest they would be 10 times more effective compared to the basic intervention. A further analysis modelled the costs per increased month of EBF due to each intervention: R337 ($48), R206 ($29), and R616 ($88) for the full, simplified and basic scenarios respectively. In addition to the average cost effectiveness the incremental cost effectiveness ratios associated with moving from the less effective scenarios to the more effective scenarios were calculated and reported: Nothing – Basic R616 ($88), Basic – Simplified R162 ($23) and Simplified – Full R879 ($126).

**Conclusions:**

The simplified scenario, with a combination of clinic and home visits, is the most efficient in terms of cost per increased month of EBF and has the lowest incremental cost effectiveness ratio.

## Introduction

Exclusive breastmilk is endorsed by the World Health Organisation as the ideal food for infants from birth to six months [1], because of its nutritional superiority over commercial formulas [2], [3], and the significant protection afforded to the infant against acute [4] and chronic illnesses [5]. Exclusive breastfeeding by HIV-infected women has recently been shown to carry less risk of postnatal HIV transmission compared to mixed feeding, particularly with solid foods [6], [7], and has been associated with greater HIV-free survival at 18 months compared to infants fed solely on formula milk [8]–[10]. There is no doubt, therefore, that exclusive breastfeeding for the first six months should be promoted globally.

Exclusive breastfeeding for six months is feasible and practical, as demonstrated in many settings [11]–[13], including high HIV prevalence areas [6]. However, most reports come from well supervised research settings, with adequate funding and personnel. Whether exclusive breastfeeding support programmes can be scaled up in operational situations, and what the financial implications of this would be to governments and health services, is questioned.

We have previously reported on a home-based counselling and support strategy to promote exclusive breastfeeding for six months in HIV-infected and uninfected women in a mostly rural setting of KwaZulu-Natal [6], [14]–[16]. We reported high rates of exclusive breastfeeding, low rates of mastitis and breast health problems, and a lower risk of postnatal HIV transmission associated with exclusive, as opposed to mixed, breastfeeding [6], [14]–[16]. This manuscript provides a costing and cost effectiveness analysis of the intervention as it was implemented, in addition to two alternative models, and estimates of the impact these would have if rolled out across the province of KwaZulu- Natal, South Africa.

## Methods

### Setting

KwaZulu-Natal is South Africa's largest province in terms of population and births. The population makes up over 20% of the country's total population, with approximately 240,000 births per year. The province is relatively balanced between urban and rural settings; the rural population comprises just over 50% of the total. KwaZulu-Natal has the highest recorded rates of HIV among women attending antenatal clinics in the country [17], [18].

### The Breastfeeding Intervention

Pregnant women attending 9 government clinics in rural, peri-urban and urban KwaZulu- Natal were enrolled into the Vertical Transmission Study (VTS) from August 2001 to September 2004; the last infant was delivered in April 2005 [6]. The study contained two components: a breastfeeding intervention strategy, designed to promote exclusive breastfeeding from birth to six months; and a research component which included documentation of feeding practices, collection of biological samples and other data. The breastfeeding intervention consisted of an antenatal and postnatal strategy and contained the following key elements:

1. Recruitment into the study at government antenatal clinic by **study HIV counsellor**
2. Group education to all pregnant women (whether enrolled or not) at antenatal clinics by **study clinic assistant**
3. Up to 4 home visits by **study lay breastfeeding counsellor** antenatally to discuss previous feeding experiences, intended feeding practices, worries or concerns, support from other family members, early positioning and attachment of the baby at the breast, and the importance of colostrum for the infant (*Antenatal breastfeeding counselling and support strategy*)
4. Postnatal visits by the **study lay breastfeeding counsellor**: 4 times in the first 2 weeks post-delivery; and fortnightly thereafter – i.e. 14 visits between birth and 6 completed months. The counsellor visited more often if the woman was experiencing difficulties or needed extra help. Postnatal visits were conducted at home in the rural/peri-urban area where 8 of the clinics were located; and with the same frequency at the clinic in the urban area (*Postnatal breastfeeding counselling and support strategy*)
5. Scheduled monthly clinic visits to **study nurses**. The objective of these visits was primarily for collection of research data. Study nurses also consolidated messages given to women by the lay counsellors and dealt with breast health problems as necessary.

All training was conducted at the site, consisting of initial infant feeding training using World Health Organization courses [19], [20]; specific training on study related issues; and further practical exercises to reinforce the training.

### The cost analysis

The costing exercise aimed to estimate the service provider costs of delivery of provincial level interventions to promote exclusive breastfeeding. Two exercises were conducted: (a) the costing of the actual intervention as it was implemented at the site; and (b) modelling the costs and outcomes associated with running such an intervention at Provincial level (KwaZulu-Natal) under three different scenarios: full intervention as per protocol, simplified version, and basic version. The full scenario examines the costs and outcomes associated with all intervention aspects of the VTS (research costs excluded) if they were implemented at provincial level. The simplified scenario is based on the same design and examines the same implications, but with a less intense design. The basic scenario examines the costs and outcomes of a substantially scaled down version of the intervention. The analysis provides estimates of the total cost of implementing each scenario and an indication of what outcome, in terms of exclusive breastfeeding, one might expect in return for such an investment compared to the outcomes you would expect from doing nothing extra. Collectively, these two outputs provide the basis for a cost effectiveness analysis (CEA) of the three scenarios. The CEA allows for the comparison not only of the total cost but also of the cost of each month of exclusive breastfeeding resulting from the programme. In addition to examining the average cost effectiveness of each programme, the incremental cost effectiveness ratios for non-dominated strategies are reported to allow for a discussion of the returns to increased investment.

The three scenarios are as follows:

### Full scenario

This essentially examines the costs and likely outcomes of implementing an intervention similar to that implemented in the VTS, with only relatively small changes.

### Simplified scenario

This is based on a similar model as implemented in the VTS, but with less frequent pre-and post-natal visits, and more clinic-based as opposed to home-based visits.

### Basic scenario

This scenario is entirely clinic-based, although it is envisaged that, as a complement, community health workers could support the intervention.

For the first two scenarios, urban and rural areas were modelled to receive different services, in line with the VTS where the intervention was largely home-based in rural areas, but clinic-based at the urban site. The basic scenario is clinic-based in both settings (Table 1).

**Table 1:** Details of the interventions by scenario and setting

	S 1 – Full scenario		S 2 – Simplified scenario		S 3 – Basic scenario	
	Urban	Rural	Urban	Rural	Urban	Rural
**Extended post-test counselling (mins)**	30	30	30	30	30	30
**Home visits (n)**						
Antenatal	0	4	1	1	0	0
Post-natal first month	0	4	1	2	0	0
Second month onward	0	2.4	0	1.2	0	0
**Clinic visits (n)**						
Antenatal	4	0	2	2	1	1
Post-natal first month	4	0	1	0	1	1
Second month onward	2.4	0	1.2	0	1	1
**Length of post-natal intervention (months)**	6	6	6	6	6	6

The scenarios differed not only in the services provided, but also in the management structure deemed necessary for implementation. The VTS was a research study with a very closely managed intervention. This management structure is modelled in the full scenario but reduced to a more reasonable level in the simplified and basic scenarios. The management structure was based on a fixed high level management and on ratios of management levels to other personnel. These ratios are provided in the annex. For the full scenario these ratios were very low compared to the other two.

The scenarios are based on modelled costs. As far as possible the resource requirement data is based on information collected at the site in order to link the results with a real intervention. The approach of using trial based data as the basis for modelled costs is common-place, allowing for provision of real data for the models [21], [22]. Collection of the site data took place during a 5-day field visit to the research site in November 2006. Financial records were reviewed and key personnel involved in VTS were interviewed, including staff at all levels: field-based breastfeeding counsellors, clinic-based staff, research staff. To supplement results of the financial data review, government employees, based at a sample of four clinics from where the VTS enrolled women, were also interviewed. The primary purpose was to obtain information on how to classify costs into research and intervention components of the study. The modelled scenarios were based on a large scale intervention so it was necessary to remove the costs of resources which were related to research only. Where data collection did not provide sufficient detail to complete the costing, further information was obtained from the coordinator of the VTS. Much of this work took place by electronic correspondence.

The financial costs of the intervention were documented retrospectively based on detailed financial records compiled by the Africa Centre's Finance Department. Only costs included as budgetary expenses were taken into account.

### Model structure

**Figure 1:**
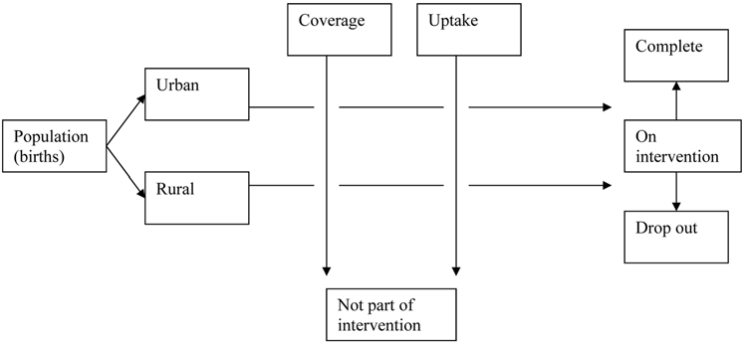
Intervention entrants.

To estimate the costs and outcomes at the provincial level it was necessary to estimate the number of women and children who would be reached by the intervention at both urban and rural sites. The number of births, by setting, in the province was taken as a starting point. This was then adjusted according to the estimated coverage of the intervention, which is itself determined by the coverage of the state sector and assumed reach of the intervention. The population entering the intervention was then further adjusted according to assumptions regarding uptake (Figure 1).

**Figure 2:**
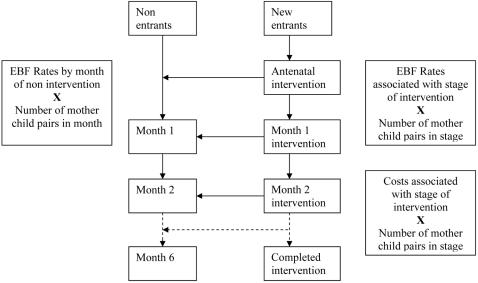
Activities and outcomes.

A monthly cycle was used to run the model, estimating the number of new entrants into the intervention under each scenario per month. Once the monthly entrant numbers were modelled, it was necessary to examine the pass through rates from month to month. Figure 2 depicts stage 2 of the modelling process. New entrants were modelled as being part of the antenatal intervention. Thereafter, the cohort of new entrants passes from month to month, with some, depending on assumptions, passing on to the non-intervention side of the model. A seven-month period was modelled for each of the scenarios to reach numbers at scale. As mother-child pairs can remain in the programme for a maximum of seven months the total number of mother-child pairs in the intervention increases in the model until the seventh month and from there on the numbers remain constant. Therefore the figures for the seventh month provide an estimate of the number of mother-child pairs who will be involved, per month, once the intervention is running at scale. The model then provides an estimate of the numbers by month, split between the intervention and non-intervention sides. The estimated effectiveness of each month was then combined with these figures to provide estimated outcomes. Similarly, the resources necessary to provide this level of service were estimated and costs attached, essentially using an activity based costing approach.

In addition to running the model for the three scenarios, it was also run as if there were no intervention. This was used as the base case. The outcome in terms of months of exclusive breastfeeding (MEBF) from this base model was subtracted from the MEBF modelled under each of the scenarios to identify the likely increase. Furthermore, in relation to effectiveness, the conservative assumptions that there would be no spillover of exclusive breastfeeding into the non-intervention population, and that dropouts would have no higher than the pre-intervention exclusive breastfeeding rates, were made for all scenarios. For the incremental cost effectiveness analysis the base case is used as the comparator for the basic scenario. The base case is assumed to carry no costs. What the incremental analysis examines is the cost of additional months gained as a result of implementing the basic scenario as opposed to doing nothing which is modeled as the base scenario. The analysis then continues to examine the additional costs compared to the additional benefits of improving this intervention to the level of the simplified scenario and then the full scenario.

The above model only considers costs, but there may also be cost savings. Exclusive breastfeeding may reduce demand for other services, or demand for free formula. Recently, there have been studies that have sought to offset these costs; these are, however, largely inappropriate. The reduced demand for other services would free up these services for other uses, which, from an economic point of view is a saving, particularly if they are re-allocated for other purposes. There is, however, no guarantee that this will happen. Furthermore, the presentation of results would be complicated by this adjustment, as it would no longer represent the budgetary implications of the interventions. There may, however, be direct savings that would have direct budgetary savings, such as reduced formula feeding. In KZN, however, formula is also provided to HIV-positive mothers after they have completed a period of breastfeeding [23]. If this policy were continued there would be no direct savings. While not included, it should be kept in mind that exclusive breastfeeding might free up other resources.

### Costs

The above model provides the numbers with which the costs and outcomes can be associated. In such exercises it is important to be clear on the costs and outcomes considered, as there are a range of possibilities. The choice of what is included is based on the purpose of the analysis; in this case to provide evidence to support the decision process regarding the introduction of a programme to support exclusive breastfeeding. There are many possible costs that could be included: provider, client and social costs [24]. From a theoretical perspective, it is most appropriate to consider social costs as, arguably, this is what should be considered by policy makers. If social, and indeed client, costs are likely to differ considerably across scenarios, or in the absence of intervention, then it is necessary to consider them [24]. This is unlikely to be the case here, except that clinic visits place a greater cost on the client compared to home visits. Given that such costs are difficult to determine, that policy options are generally presented with only provider costs, and that social costs would complicate comparison, the decision to consider only provider costs was taken. The costs reported, therefore, only reflect those incurred by the health care system.

As far as possible, cost data were obtained from the VTS site. Mainly resource use data were taken from the site; the costs attached to these resources were drawn from provincial data. For example, data on time spent by staff on different tasks were drawn from the site, while the costs associated with staff at different levels were taken from the provincial human resources scales [25]. For structures, such as provincial management, required at scale but not required at site level, data from similar existing programmes – namely, the provincial prevention of mother-to-child transmission (PMTCT) interventions - were used with adjustments for scale. Given the nature of the intervention it bears many similarities to the PMTCT intervention in terms of its location in antenatal and baby clinics, and its mixed use of medical professionals and lay staff. Given these similarities it is reasonable to assume a similar management structure.

### Outcomes

Exclusive breastfeeding is a critical factor in child survival and carries less risk of postnatal HIV transmission compared to mixed feeding. There are, therefore, a variety of potential outcome measures that could be used. For the purposes of this work, the outcome measure used was months of exclusive breast feeding (MEBF), which has a number of advantages over alternatives, but also some notable disadvantages. The measure is useful as it relates to all infants in the intervention, both HIV-exposed and unexposed, whereas a measure such as HIV infections averted relates only to a sub-sample. It is also useful as the data on this outcome were collected extremely rigorously in the VTS [6]. The major drawback is that it is a very specific outcome making comparison with other types of intervention difficult. For comparisons across scenarios it is perfect, as they all have the same aim; the problem is alternative interventions to improve child health via routes other than exclusive breastfeeding as these will have different outcome measures which will make comparison impossible.

The data on exclusive breastfeeding rates associated with each scenario were based on adjusted rates from the VTS. The VTS provided detailed estimates of the rate of exclusive breastfeeding observed among study participants and how these change depending at what stage of the intervention mothers are in [15]. Data were not available on how reductions in the intensity or changes in the nature of delivery would affect outcomes. The VTS rates were therefore used as a starting point and adjustments were then made according to advice from the implementation team, who considered their experience and the data that were available. The VTS team were asked to estimate how reducing the intensity of the intervention at different points would influence outcomes. As directly relevant data were not available the implementation team's extensive experience, which included drawing on data from pilot work conducted in the area prior to the implementation of the breastfeeding strategy [26], was considered the most appropriate source of information for determining adjustments. The rates of exclusive breastfeeding observed during the study and the adjustments applied to them are presented in the annex. The estimates of reduced effectiveness were applied under two sets of assumptions: full reduction and part reduction. Under the full reduction assumption impacts of changes from the VTS protocol were considered to be cumulative across the months, whereas under the part reduction assumption they were considered to last only for the one month. For example, it was assumed that having fewer visits in the first month would lead to lower rates of EBF. Under the full reduction assumption this reduced effectiveness was carried through to every subsequent month even if the intervention was then implemented according to protocol. Under the part reduction assumption effectiveness was considered to fall only for the month of the change and if implemented according to protocol then rates would return to those observed in the study from the next month onwards. The first assumption was considered more realistic and the second assumption was only included as part of the sensitivity analysis.

The costs and outcomes estimated for each scenario are presented as totals, average cost effectiveness ratios and incremental cost effectiveness ratios. The totals reflect the total cost of implementing the scenario at a provincial level and the outcome total represents an estimate of the increase in MEBF resulting from such implementation. The cost effectiveness ratios are the average cost per additional MEBF while the incremental cost effectiveness analysis ratios are the additional cost per additional MEBF over and above that achieved by the next most effective intervention. Incremental cost effectiveness analysis is only relevant when an intervention is both more effective and more expensive. By assumption the interventions become both more costly, as they become more intensive, and more effective as one moves from the basic to the full scenario.

Cost effectiveness analysis (CEA) is a powerful tool but must be interpreted with some caution. CEA in this case will identify the relative efficiency of the alternative scenarios in generating MEBF. The results of such efficiency analysis are often interpreted as showing one intervention to be better than the others. This is not the case; CEA only shows which is more efficient and efficiency is only one criteria. Policy makers may well choose a less efficient option, spend more, but as a result generate a higher number of MEBF. For example, when finances are not a major constraint and there is full coverage, the most cost effective option may be ignored in favour of a more effective intervention so as to improve outcomes.

### Limitations

The above method was designed to generate the most useful results within the constraints of the project. The model used is a population model and, as such, relies on resource-to-client ratios to estimate costs. This ignores to some extent the distribution of demand. Assuming that a new unit of a resource is required once the last has reached capacity, implies that clients and resources can be perfectly matched, or that resource units are dividable, which may not always be the case. For example, if 100 clients required one counsellor the model would cost one counsellor. However, if those clients were divided across two clinics there may be a need for two counsellors – one in each clinic. If the counsellors could be employed part-time or could travel, this would not be a problem. This limitation has been countered to some extent by allowing for some transport costs of staff, allowing staff to travel between sites and, thus, being able to divide their time between them. An infrastructure component could be added to the model which would examine the likely demand at specific institutions, but it was felt that this would add unnecessary complexity: unnecessary because the object is to examine the costs and outcomes at scale, not in KZN in particular. KZN is the example; if this were a costing specifically for provincial planning there might be an argument for the addition.

The more fundamental limitation is the data on outcomes. The exercise requires that estimates of outcomes be made, and adjustments were made to the VTS outcome data to do so. These adjustments were, however, based on assumptions regarding the impact of different aspects of the intervention and are therefore untested. In order to be cautious, the implementation team favoured making very conservative assumptioms regarding the impact of lower intensity. The assumptions were conservative in the sense that they were not overly optimistic about the possibility of a scaled down intervention having as similar an impact as the full implementation.

Appendix S1 shows the assumptions used in the modeling exercise, including the management structure, and coverage and uptake assumptions.

### Ethical approval

The VTS study was approved by Biomedical Research Ethics Committee of the University of KwaZulu-Natal. The additional costing component was also approved by the Social Science Ethics Committee of the Human Sciences Research Council.

## Results

The following section presents the results of the analyses detailed above. Firstly, the costs are discussed, then the outcomes, and finally a combination of the two. The analysis was based on a model with monthly iterations (using the figures from the seventh month) but for the purposes of comparison with similar work, the results are presented annually. The total annual costs estimated for each scenario are presented in Table 2. The costs are broken down according to cost categories and are presented in 2007 South African Rand. The amount first mentioned of any figure is accompanied by the equivalent United States Dollar amount using an exchange rate of $1 = R7.

**Table 2:** Total annual cost by scenario

	S 1 – Full Scenario	S 2 – Simplified scenario	S 3 – Basic scenario
Compensation (%)	90	94	95
Facilities (%)	0	0	1
Equipment (%)	1	1	1
Transport (%)	7	4	3
Communications (%)	2	1	1
**Total cost (Rands)**	**95 135 729**	**46 774 845**	**13 608 971**
**Total cost (US$)**	**13 590 818**	**6 682 121**	**1 944 139**
**Incremental cost (Rands)**	**48 360 884**	**33 165 874**	**13 608 971**
**Incremental cost (US$)**	**6 908 697**	**4 737 982**	**1 944 139**

The costs of implementation of the full scenario are, as would be expected, far greater than those of the other two. It was estimated that the full scenario would cost over R95 million ($14 million) per annum. This is an estimate of what it would cost if the intervention, as it was structured in the VTS, was offered across the province of KZN. The simplified scenario came out closer to R47 million ($7 million) and the basic was by far the lowest estimate - a little over R14 million ($2 million). The incremental costs indicate the additional costs associated with moving to a more intensive intervention. The results suggest that the largest jump in costs would be associated with moving from a simplified to a full scenario.

For all three scenarios, the major cost item was compensation (i.e. salaries), which in all scenarios accounted for over 90% of the total cost; the interventions are all labour intensive. Table 3 provides the results of the modelling exercise on the staff needs associated with each scenario on which the above costs are based.

**Table 3:** Implementation staff requirements by scenario

Category of staff (n)	S 1 – Full scenario	S 2 – Simplified scenario	S 3 – Basic scenario
PMTCT^1^ counsellors	51	51	51
BF^2^ counsellor for home visits	928	448	34
BF counsellor for clinic-based visits	241	141	183
Clinic assistants	292	292	0
Supervisors	185	25	7
Managers	22	6	0
Infant feeding specialists	18	0	0

1 Prevention of mother-to-child transmission counsellor.

2 Breastfeeding counsellor.

All three scenarios involved extended PMTCT counselling to introduce the intervention and so have similar requirements in this regard. The more intense the intervention the more breastfeeding counsellors would be needed. In view of the intensity of the full and simplified interventions, a clinic assistant was included to support the intervention; this position was not deemed necessary in the basic scenario. The management ratios modelled in the full scenario were considered unnecessarily high, and thus were reduced in the other scenarios. This, combined with fewer counsellors, resulted in an estimate of a far smaller number of supervisors and managers needed in the second two scenarios. In scenario 2, the roles of infant feeding specialist and manager were combined and included only under the manager heading. There is a significant demand for labour across the scenarios, although obviously more so in the first two. It is, however, important to note that the major demand is for counsellors and clinic assistants and not health professionals.

Starting up interventions requires training for the new staff. These training costs were estimated on the basis of the modelled staff needs and amounted to R2.1, R1.2, and R0.3 million for scenarios 1, 2 and 3 respectively ($300, $170 and $48 thousand respectively). Obviously, the fewer staff requiring training the lower the costs predicted, resulting in far larger training cost estimates for scenarios 1 and 2 compared to 3.

Thus far the results show that the full scenario is the most expensive of the three options, designed to lead to the same type of outcome. This is because it is more intensive and would be expected to lead to better outcomes. Table 4 presents the results of the estimated impact of the three interventions. The impact is reported in three forms: firstly, the total number of months women were supported to exclusively breastfeed (Supported MEBF); secondly, an estimate of the number of MEBF which would have occurred in the absence of intervention is subtracted from the estimate of supported months to provide an estimate of the number of those months that are a result of the intervention and would not have occurred otherwise (Increased MEBF). Finally the incremental increase in MEBF which result from moving from no intervention to basic, from basic to simplified, and from simplified to full, is reported. These final figures indicate how much return is generated as a result of the investment in a more intensive, as opposed to a less intensive, intervention.

**Table 4:** Outcomes and cost effectiveness results

	S 1 – Full scenario	S 2 – Simplified scenario	S 3 – Basic scenario
**Outcomes – full reduction**			
Supported MEBF*	330 220	275 223	69 771
MEBF with no intervention	48 273	48 273	48 273
Increased MEBF	281 947	226 950	22 306
Incremental increase in MEBF	54 997	204 644	22 306
**Cost effectiveness ratios – full reduction**			
Cost per supported MEBF	R288 ($41)	R170 ($24)	R195 ($28)
Cost per increased MEBF	R337 ($48)	R206 ($29)	R616 ($88)
Incremental cost effectiveness	R879 ($126)	R162 ($23)	R616 ($88)
**Cost effectiveness ratios – part reduction**			
Cost per supported MEBF	R288 ($41)	R148 ($21)	R54 ($8)
Cost per increased MEBF	R337 ($48)	R175 ($25)	R66 ($9)
Incremental cost effectiveness	R6 448 ($921)	R769 ($110)	R66 ($9)

* MEBF = months of exclusive breastfeeding.

The modelled effectiveness of the more intensive scenarios (full and simplified) suggested that these interventions would be more than 10 times as effective than the basic intervention in increasing the number of months of exclusive breastfeeding. So, while the basic scenario is far cheaper, it is also predicted to be far less effective. These effectiveness estimates were based on the assumption that changes at any stage of the original intervention would impact on effectiveness for the balance of the intervention not only in the period of the change. Results based on this assumption are labeled full reduction.

Considering both the costs and outcomes together allows for the examination of the relative efficiency of the three options under consideration. Table 4 also provides three cost/outcome ratios. The first is the total cost divided by the number of months women who participated in the intervention breastfed exclusively for (Cost per supported MEBF). The second is the cost divided by the months of exclusive breastfeeding that occurred only as a result of the intervention (Cost per increased MEBF). The third is the incremental cost effectiveness ratios. This final ratio reports the increase in costs divided by the increase in MEBF as a result of moving from a less to a more intensive intervention. In the case of the basic scenario the comparison is with doing nothing which has no cost but would still lead to some MEBF.

Examining first the cost per supported MEBF: despite the higher effectiveness, the full scenario is the most expensive per unit; the simplified is the most efficient, with the basic a close second. The basic, however, is a close second only because in this measure it is given credit for supporting mothers who exclusively breastfed even if they would have done so anyway. Once the interventions are evaluated in terms of the cost per increased MEBF the simplified scenario is by far the most efficient. The basic scenario, while much cheaper, was predicted to be so ineffectual that it is estimated to be very inefficient. The full scenario, on the other hand, was predicted to be more effective but also much more costly and so also less efficient. The incremental cost effectiveness ratios highlight this point. The additional MEBF which result from moving from nothing are, under the full reduction assumption, very expensive at R616 per month, while the months gained from moving from the basic to the simplified are relatively much cheaper at R162 per month. It is this improved efficiency which drives down the average cost effectiveness ratio. Continued increases in intensity, however, do not show the same trend. The high cost of the additional months gained as a result of moving to the full scenario push the average cost up suggesting the setting in of diminishing marginal returns.

The above discussions focus on the results based on the full reduction assumption. It could be argued that this assumptions is too harsh. By way of sensitivity analysis the following is provided at the other end of the spectrum. As mentioned, the results above were based on the assumption that changes at any point in the intervention alter outcomes from there on. For this reason changes in the antenatal part of the breastfeeding counselling and support intervention had the greatest impact on outcomes. Antenatally, women received up to 4 home visits by breastfeeding counsellors where discussions about previous feeding experiences, how to breastfeed after delivery and support from the rest of the family were discussed. Table 4 also reports the cost effectiveness results based on the assumption that changes only affect one month and the intervention returns to full effectiveness thereafter. Results based on this assumption are labeled ‘part reduction’. In this model antenatal changes only affect the first month of feeding. This is not presented as a realistic assumption, but rather to show the sensitivity of the results to assumptions regarding changes in the effectiveness resulting from changes in the design. With the lack of antenatal services in the basic scenario no longer dominating the results, it becomes the most effective by some distance.

## Discussion

The Vertical Transmission Study (VTS) was highly successful in promoting optimal feeding practices amongst HIV-infected and uninfected women in a mostly rural South African setting, using a model based on lay counsellors [6], [14]–[16]. Whether this intervention could be replicated on a larger scale, and what this would cost to the province of KwaZulu-Natal, are questions which have been raised since our results have been published. It is clear that the highly intensive nature of the VTS strategy would make the scaling-up of an identical intevention to the entire province very expensive. Therefore, we have costed not only a replication of the full VTS breastfeeding intervention but also two alternative simpler interventions.

The full intervention would cost the Province approximately R95 million per annum to implement. Whereas the basic model (R14 million per annum) is unlikely to have a great impact on adherence to exclusive breastfeeding, the CEA suggests that the simplified model (R47 million per annum) would be the most efficient option. No intervention is dominated by another as each increase in cost is also associated with an increase in effectiveness. The returns to increasing costs do, however, vary dramatically. The incremental cost effectiveness analysis highlights the economies of intensity of intervention as well as the occurrence of diminishing marginal returns. Increasing the intensity of the intervention from basic to simplified pulls down the average cost effectiveness as a result of the low incremental cost effectiveness suggesting an improvement in efficiency. However, this result does not follow through to a shift to the full scenario as the incremental cost effectiveness analysis suggests that the marginal cost of MEBF would increase rapidly and climb above the average cost thereby pulling it up. Nevertheless, the results of the CEA should be interpreted with caution, and not taken to clearly recommend one scenario over the others. If a province such as KwaZulu-Natal were deciding between the three scenarios, the results would recommend that the full scenario is chosen if they wish the results of the strategy to result in as many months of exclusive breastfeeding as possible, and if cost was not an issue. If the province wished to have as high coverage as possible for as low a cost as possible, and the outcome itself was not the primary factor, then our results would suggest pursuing the basic scenario. However, if the province wanted to promote exclusive breastfeeding but had a limited budget that was less than the total needed for the full scenario, they should pursue the second option – the simplified version. This last set of circumstances is typically the most common, which is why the results of the CEA are usually interpreted as making a clear recommendation, but it is worth noting that the recommendation is only valid in these particular circumstances. It may, however, also be that in certain areas there may be greater need for improvements in rates of exclusive breastfeeding, possibly because current rates are even lower than elsewhere or HIV prevalence is particularly high. In these settings the cut off in terms of the cost of an additional month which the health system would be willing to carry may be higher and therefore they may consider a more intensive intervention.

As mentioned previously, one of the circumstances in which the CEA results do lead to clear recommendations is the acceptance that exclusive breastfeeding is desirable, given the cost. The above results provide some support to policy makers wishing to make a decision in this regard. If the benefits of a month of exclusive breastfeeding are worth more than R206 then the intervention is worthwhile, while if the value is greater than R879 then it is worth intensifying the intervention and moving towards the full scenario. Attaching a rand value to benefits such as child health is extremely controversial and based largely on value judgements; as a result so, too, is the decision on how much to invest in exclusive breastfeeding. Typically in cost effectiveness analysis, ratios are compared to threshold values in terms of, for example, cost per DALY or QALY. Unfortunately, there are not such thresholds relating to MEBF.

It should be emphasized that this intervention was designed to promote optimal feeding practices amongst all women, not only those who were HIV-infected. Despite the high HIV prevalences amongst pregnant women in KwaZulu-Natal, the majority of pregnant women in South Africa are HIV-uninfected. It is crucial for overall child health in the country that HIV-uninfected women are encouraged and supported to exclusively breastfeed for the first six months of life, with continued breastfeeding after the introduction of complementary feeds for at least two years [1]. There is evidence that unclear and mixed messages around formula feeding in PMTCT programmes is resulting in a spill-over of sub-optimal infant feeding practices to HIV-uninfected women [27], [28]. The investment made to promote overall optimal infant feeding practices (R95 million per annum for the total intervention in the province) should be viewed in light of the advantages gained by all children, irrespective of the HIV status of their mothers.

This model would not require significant numbers of additional professional nurses as it was based on carefully selected and trained lay counsellors from the local communities, which is particularly attractive given the high unemployment rates in rural areas. Furthermore, the model is generalisable and could be scaled-up using existing lay workers in the province, for example community health workers, to promote and support exclusive breastfeeding within the families they already visit as part of their routine work. The training was based on standard WHO courses [19], and the recruitment, training and support of the counsellors has been well documented. Promoting and supporting exclusive breastfeeding has financial implications, and policy makers need to decide how much they wish to invest in this critical element of child health [29], [30].

## Supporting Information

Appendix S1 (0.06 MB DOC)
