# Supplementary material for: Scaling-Up Exclusive Breastfeeding Support Programmes: The Example of KwaZulu-Natal
Source: PLoS One. 2008 Jun 18;3(6):e2454. doi: 10.1371/journal.pone.0002454 (PMC2413404; doi:10.1371/journal.pone.0002454)
Supplement: Appendix S1 — (0.06 MB DOC) [file pone.0002454.s001.doc]

**Appendix S1: Model data and assumptions**

The following three tables present key data used and assumptions made in the modelling exercise. The first table details the assumptions regarding the management structure, the provincial context and coverage and uptake assumptions.

The management structure is divided into two parts. Provincial management refers to the semi-fixed management structures based on comparisons to the PMTCT provincial programme which is scaled down only for the basic scenario. The second part relates to variable management structures linked to coverage. The model was used to estimate the number of counsellors required based on the intensity of the visiting and then the following ratios were applied to estimate the number of management staff required as a result.

**Table A1**: Key model assumptions

|  | S 1 – Full | S 2 - Simplified | S 3 – Basic |
| --- | --- | --- | --- |
| Provincial management |  |  |  |
| Director | 1 | 1 | 1 |
| Deputy director | 2 | 2 | 2 |
| Regional | 5 | 5 | 2 |
| Administration | 3 | 3 | 1 |
|  |  |  |  |
| Ratios of field management to other staff |  |  |  |
| Home based counsellor/supervisor | 6 | 24 | 30 |
| Clinic based counsellor/supervisor | 8 | 24 | 30 |
| Supervisor/manager | 9 | 30 | 30 |
| Infant feeding specialist/supervisor | 10 | - | - |
|  |  |  |  |
| Birth per annum in province | 240 000 | 240 000 | 240 000 |
| Proportion urban | 0.4 | 0.4 | 0.4 |
| Coverage of state facilities | 0.9 | 0.9 | 0.9 |
| Coverage of intervention | 0.9 | 0.9 | 0.9 |
| Portion uptake of intervention | 0.5 | 0.5 | 0.5 |
| Monthly drop out (clinic based) | 0.05 | 0.05 | 0.05 |
| Monthly drop out (home based) | 0.03 | 0.03 | 0.03 |

The effectiveness of the intervention was based on data drawn from the VTS. The following table outlines the rates of exclusive breast feeding expected with no intervention and with the implementation of the full intervention. The rates differ for HIV-positive and HIV-negative mothers given the ongoing feeding choices HIV-positive women are expected to make, and the necessity of ceasing breastfeeding by 6 completed months after delivery and switching to non-human milk.

**Table A2:** Rates of exclusive breastfeeding by month*

| Months | No intervention | | Full intervention | |
| --- | --- | --- | --- | --- |
|  | HIV+ | HIV- | HIV+ | HIV- |
| EBF at 1 month | 0.16 | 0.16 | 0.83 | 0.82 |
| EBF at 2 month | 0.1 | 0.1 | 0.74 | 0.76 |
| EBF at 3 month | 0.08 | 0.08 | 0.67 | 0.69 |
| EBF at 4 month | 0.08 | 0.08 | 0.6 | 0.64 |
| EBF at 5 month | 0.08 | 0.08 | 0.54 | 0.58 |
| EBF at 6 month | 0.08 | 0.08 | 0 | 0.53 |
| EBF at 6 months + | 0.08 | 0.08 | 0 | 0.49 |

*Source: VTS

The results in the above table were adjusted following discussions with the implementation team. Given their vast experience in implementing such programmes they were deemed to be the best source of information on how reduced intensity would impact on outcomes[26]. The table provides the assumptions regarding reductions in effectiveness of the full intervention associated with changes in the protocol.

**Table A3**: Outcome adjustments

| Aspect | Adjustment | Reduction in effectiveness |
| --- | --- | --- |
| Antenatal visits | 1 | 0.9 |
|  | 2 | 0.5 |
|  | 3 | 0 |
|  | 4 | 0 |
|  |  |  |
| First month | 1 | 0.25 |
|  | 2 | 0.25 |
|  | 3 | 0 |
|  | 4 | 0 |
|  |  |  |
| Post-natal per 4 weeks | 2 | 0 |
|  | 1 | 0 |
|  | 0.5 | 0.25 |
|  | 0 | 0.9 |
|  |  |  |
|  |  |  |
| Time per visits | 45 | 0 |
|  | 30 | 0 |
|  | 15 | 0.2 |
|  | 5 | 0.75 |

Unit cost data are not reported as they consist almost exclusively of staff costs which were provided by the Department of Health on condition that the unit costs not be published.
